# VEGF Signaling through Neuropilin 1 Guides Commissural Axon Crossing at the Optic Chiasm

**DOI:** 10.1016/j.neuron.2011.02.052

**Published:** 2011-06-09

**Authors:** Lynda Erskine, Susan Reijntjes, Thomas Pratt, Laura Denti, Quenten Schwarz, Joaquim M. Vieira, Bennett Alakakone, Derryck Shewan, Christiana Ruhrberg

**Affiliations:** 1 School of Medical Sciences, Institute of Medical Sciences, University of Aberdeen, Aberdeen, AB25 2ZD, UK; 2 Genes and Development Group, Centres for Integrative Physiology and Neurosciences Research, University of Edinburgh, Edinburgh, EH8 9XD, UK; 3 UCL Institute of Ophthalmology, University College London, London, EC1V 9EL, UK

## Abstract

During development, the axons of retinal ganglion cell (RGC) neurons must decide whether to cross or avoid the midline at the optic chiasm to project to targets on both sides of the brain. By combining genetic analyses with in vitro assays, we show that neuropilin 1 (NRP1) promotes contralateral RGC projection in mammals. Unexpectedly, the NRP1 ligand involved is not an axon guidance cue of the class 3 semaphorin family, but VEGF164, the neuropilin-binding isoform of the classical vascular growth factor VEGF-A. VEGF164 is expressed at the chiasm midline and is required for normal contralateral growth in vivo. In outgrowth and growth cone turning assays, VEGF164 acts directly on NRP1-expressing contralateral RGCs to provide growth-promoting and chemoattractive signals. These findings have identified a permissive midline signal for axons at the chiasm midline and provide in vivo evidence that VEGF-A is an essential axon guidance cue.

## Highlights

► NRP1 is expressed by contralateral mouse retinal ganglion cell axons ► NRP1 promotes commissural axon crossing at the optic chiasm as a VEGF164 receptor ► VEGF164 acts directly on retinal growth cones as an attractive guidance signal ► VEGF164 and NRP1 are essential for normal contralateral axon growth in vivo

## Introduction

Retinal ganglion cells (RGCs) relay visual information from the eye to the higher visual processing centers of the brain in all vertebrates. They do so by extending axons through the optic disc into the optic nerve and then projecting to their primary target, the superior colliculus in mammals. En route, they pass through the diencephalon, forming a major commissure known as the optic chiasm. In vertebrates with frontally located eyes, subpopulations of RGC axons segregate at the optic chiasm to project to targets on both the ipsilateral and contralateral sides of the brain to establish binocular vision (reviewed by Erskine and Herrera, 2007; Petros et al., 2008). In species with a small overlap in the visual field—for example, mice—the vast majority of RGCs projects contralaterally, with ipsilaterally projecting RGCs comprising only ∼3% of the total RGC population. Most ipsilateral RGCs originate in the ventrotemporal crescent of the mouse retina, where they are specified by the zinc-finger transcription factor ZIC2 (Herrera et al., 2003). The defined origin and stereotypical behavior of the contralaterally and ipsilaterally projecting RGC axons has made the optic chiasm an important model system for the study of axon guidance (reviewed by Erskine and Herrera, 2007; Petros et al., 2008).

A collection of in vitro and in vivo studies suggests that the midline environment of the diencephalon is inhibitory to RGC axon extension (Godement et al., 1994; Wang et al., 1995, 1996; Mason and Wang, 1997). Accordingly, several repulsive cues cooperate to repel the growth cones of RGC axons at the optic chiasm (reviewed by Erskine and Herrera, 2007). These include SLIT proteins to define the boundary of the optic pathway (Plump et al., 2002), and ephrin B2, which is a midline repellent for RGC axons destined for the ipsilateral optic tract (Nakagawa et al., 2000; Williams et al., 2003). The only factor known to promote axon crossing at the chiasm is the cell adhesion molecule NrCAM (Williams et al., 2006). Even though NrCAM is expressed at the chiasmatic midline, it does not serve as a guidance cue; rather, it is required cell autonomously in the axons of a small subset of late-born RGCs to promote their contralateral projection, perhaps as a receptor for attractive ligands (Williams et al., 2006). Thus far, no midline factor has been identified that is required for RGC axons to project contralaterally.

In the search for molecules that regulate axon divergence at the optic chiasm in mammals, we investigated two members of the neuropilin family, NRP1 and NRP2 (reviewed by Schwarz and Ruhrberg, 2010). These transmembrane proteins contribute to many aspects of nervous system wiring by serving as receptors for axon guidance cues of the class 3 semaphorin (SEMA) family. Moreover, mouse RGCs express NRP1 when they are growing within the brain, and express NRP2 at least during postnatal development (Kawakami et al., 1996; Gariano et al., 2006; Claudepierre et al., 2008). Studies in zebrafish suggest that the NRP1 ligand SEMA3D provides inhibitory signals at the chiasm midline to help channel RGC axons into the contralateral optic tract (Sakai and Halloran, 2006). However, the functional significance of neuropilin expression for RGC axon guidance at the mammalian optic chiasm has not been determined. Moreover, the possible role of VEGF164, a neuropilin ligand that is structurally distinct from SEMAs, has not been considered previously in any studies of pathfinding in the visual system.

VEGF164, known as VEGF165 in humans, is an isoform of the vascular endothelial growth factor VEGF-A (Soker et al., 1996). It is best known for its ability to stimulate endothelial cell proliferation and migration during blood vessel growth, but has more recently been proposed to also promote neural progenitor proliferation, differentiation, and survival (Robinson et al., 2001; Hashimoto et al., 2006; reviewed by Ruiz de Almodovar et al., 2009). In vitro, VEGF-A promotes axon outgrowth of various neuronal cell types, for example, during the regeneration of postnatal RGCs (Böcker-Meffert et al., 2002). However, it is not known if this is a direct effect on axon guidance or if this is due to increased cell proliferation or survival in the cultured tissue. To date no study has identified an in vivo role for VEGF in axon guidance.

To determine if neuropilins regulate RGC pathfinding in mammals, we delineated their expression patterns in the developing mouse optic pathway and combined genetic analyses with in vitro models to study their contributions to RGC axon guidance. We found that NRP1, but not NRP2, was expressed by RGC axons as they extended through the optic chiasm, and that NRP1 was required by a subset of RGC axons to project contralaterally. Unexpectedly, this essential role for NRP1 in chiasm development was due to its ability to serve as a receptor for VEGF164 rather than SEMAs. Thus, loss of VEGF164 and NRP1, but not class 3 SEMA signaling through neuropilins, increased ipsilateral projections at the expense of contralateral projections. This requirement of VEGF164 for contralateral guidance at the chiasm was independent of VEGF-A's role in blood vessels, and was due to its ability to act as a growth-promoting factor and chemoattractive cue for NRP1-expressing RGC axons. Beyond their significance for understanding axon wiring in the visual system, these findings provide evidence that VEGF-A is a physiological axon guidance cue with a key role in commissural axon guidance.

## Results

### NRP1 Is Expressed by Mouse RGCs

**Figure 1:**
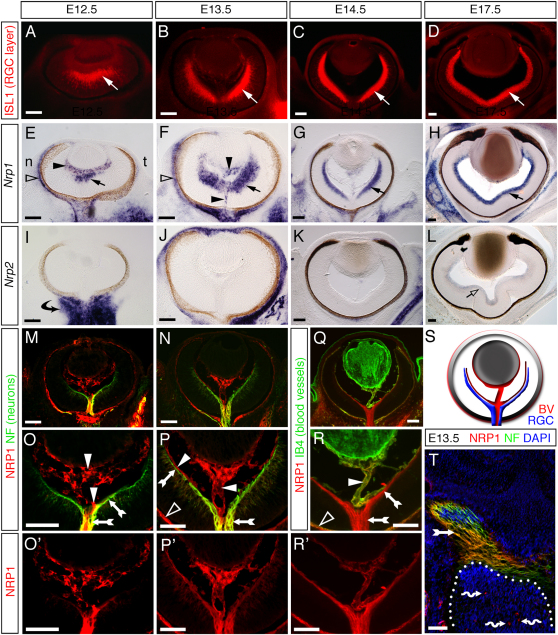
Mouse RGCs Express NRP1, but Not NRP2, When Their Axons Cross the Optic Chiasm (A–L) Immunofluorescence labeling (A–D) and in situ hybridization (E–L) of horizontal sections through wild-type eyes at E12.5–17.5, the time when RGCs differentiate and extend axons through the optic chiasm. ISL1 staining (A–D) illustrates the position of RGC neurons (white arrows). *Nrp1* (E–H) is expressed in the RGC layer (solid arrows) and by hyaloid and choroidal vessels (solid and clear arrowheads, respectively). In contrast, *Nrp2* (I–L) is expressed in mesenchyme surrounding the eye (curved arrow in I), but not in blood vessels; expression in the RGC layer begins only at E17.5 (clear arrow). (M–R) Double immunofluorescence staining of horizontal sections through the eye with antibodies specific for NRP1 (red) and neurofilaments (NF; green in M–P) or IB4 (green in Q and R). Yellow staining indicates colocalization. NRP1-positive RGC axons are indicated with feathered arrows; hyaloid vessels, with solid arrowheads; and choroidal vessels, with clear arrowheads. (O) and (O′), (P) and (P′), and (R) and (R′) are higher magnifications of (M), (N), and (Q), respectively. (S) Schematic relationship of NRP1-positive blood vessels (BV) and RGC axons in the developing eye. (T) Double immunofluorescence staining of a horizontal section through the optic chiasm with antibodies specific for NRP1 (red) and neurofilaments (NF; green); the section was counterstained with the nuclear marker DAPI (blue). Feathered arrows indicate RGC axons; wavy arrows, capillaries in the diencephalon (outlined with a white dotted line). Scale bars: 100 μm.

We found that mouse RGCs expressed NRP1 throughout the period of optic chiasm development (Figure 1). We first compared the expression of *Nrp1* to that of ISL1, a marker for the RGC layer (Figures 1A–1D). *Nrp1* mRNA was expressed strongly in the central region of the E12.5 retina (Figure 1E), where the first RGCs are born (Figure 1A; Godement et al., 1987). At E13.5, *Nrp1* expression extended peripherally, correlating with the pattern of RGC generation (Figures 1B and 1F). At E14.5, *Nrp1* was expressed throughout the RGC layer (Figure 1G), where it continued to be expressed strongly until at least E17.5, the latest age examined (Figure 1H). The hyaloid vasculature also expressed *Nrp1* (Figures 1E and 1F, black arrowheads), like other blood vessels in the central nervous system (Kawasaki et al., 1999; Fantin et al., 2010). In contrast, *Nrp2* expression was not detected in the retina until E17.5 (Figures 1I–1L), when the majority of axons have already navigated through the optic chiasm (Godement et al., 1987). Instead, *Nrp2* was expressed strongly by mesenchyme surrounding the developing optic nerve (Figure 1I, black arrow).

Double immunofluorescence staining of sections with a highly specific antibody for NRP1 (Fantin et al., 2010) and antibodies for neurofilaments or the blood vessel marker isolectin B4 (IB4) confirmed that NRP1 protein was expressed by RGCs (Figures 1M–1S). They also revealed that NRP1 localized predominately to RGC axons in the optic fiber layer at the inner surface of the retina, rather than RGC bodies within the retina (Figures 1O, 1O′, 1P, 1P′, and 1R′). NRP1 was also prominent on RGC axons projecting through the optic chiasm (Figure 1T). Finally, double labeling with antibodies for BRN3A (POU4F1), a transcription factor expressed by RGCs (Xiang et al., 1995), demonstrated that NRP1-positive axons emerged from the RGC layer (Figure S1 available online). We conclude that NRP1, but not NRP2, is expressed in the developing mouse visual system at the correct time and in the right place to play a role in RGC axon growth.

### NRP1 Regulates Axon Crossing at the Optic Chiasm

**Figure 2:**
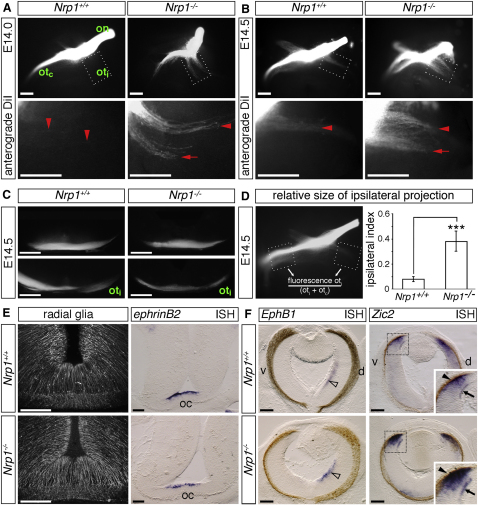
NRP1 Is Essential for Normal Optic Tract Organization and Contralateral Projection at the Optic Chiasm (A and B) Wholemount views of RGC axons at the optic chiasm, labeled anterogradely with DiI at E14.0 (A) and E14.5 (B) in littermates expressing or lacking NRP1; ventral view, anterior up (see Figure S2A). The optic nerve (on), contralateral optic tract (ot_c_), and ipsilateral optic tract (ot_i_) are labeled in the first wild-type panel. Boxed regions are shown at higher magnification below each panel. Red arrowheads indicate the normal position of the ipsilateral projection; red arrows, the secondary tract and axon defasciculation in the mutants. (C) Coronal sections through the optic chiasm (top panels) and the site where the optic tracts begin to diverge (bottom panels) of anterogradely labeled E14.5 *Nrp1^+/+^* and *Nrp1^−/−^* brains. (D) Ipsilateral index in *Nrp1* null mutants. The method used to determine the ipsilateral index is shown on the left-hand side (see Supplemental Experimental Procedures for details). The mean (±SEM) ipsilateral index of E14.5 *Nrp1^+/+^* and *Nrp1^−/−^* littermates is shown on the right-hand side; n = 10 each; ^∗∗∗^p < 0.001 compared to wild-types. (E) Immunofluorescence labeling of radial glia and in situ hybridization (ISH) for *ephrinb2* in coronal sections through the optic chiasm (oc) of E14.5 littermates expressing or lacking NRP1; dorsal is up. (F) ISH of coronal sections through stage-matched eyes expressing or lacking NRP1. *Ephb1* identifies early ipsilaterally projecting RGCs in the dorsocentral retina (clear arrowhead). *Zic2* identifies permanent ipsilaterally projecting RGCs in the ventrotemporal retina; the area outlined with a dotted square is shown at higher magnification in the insets; arrows indicate *Zic2*-positive RGCs; arrowheads, the ciliary margin. d, dorsal; v, ventral. Scale bars: 250 μm (A–C); 120 μm (E and F).

To determine if NRP1 is essential for RGC pathfinding at the optic chiasm, we studied mice carrying a *Nrp1* null mutation on a mixed CD1/JF1 genetic background, which ameliorates the severe cardiovascular defects seen in mutants on the C57 BL/6J background and enables embryo survival until E14.5 (Schwarz et al., 2004). We performed anterograde DiI labeling of RGC axons from one eye at E14.0, when axons have just entered the optic tracts, and at E14.5, when both contralateral and ipsilateral tracts are established (Figure S2A). Wholemount views of the chiasm revealed striking and consistent differences in RGC organization between homozygous mutants and their wild-type littermates (Figures 2A and 2B; n = 10 each). First, all mutants showed defasciculation of both the ipsilateral and contralateral optic tracts, with axons being organized into two discrete bundles. Consequently, the normal asymmetry in the width of the contralateral and ipsilateral tracts was lost in the mutants. Second, the proportion of axons projecting ipsilaterally appeared increased in the mutants.

Sections through the DiI-labeled brains showed that the optic tracts were thinner in mutants than in wild-types, due to their defasciculation (Figure 2C). However, the path taken by the mutant axons appeared normal, both at the level of the optic chiasm (Figure 2C, top panels) and at the site where the optic tracts began to diverge (Figure 2C, bottom panels). Thus, axons did not stray from the pial surface or project aberrantly at the midline, as seen in mutants lacking SLITs (Plump et al., 2002). Gross disturbances in axon guidance at the midline are therefore not the likely cause of the increased ipsilateral projection in *Nrp1* null mutants.

Owing to the lethality of *Nrp1* null mutants at E15.5, we could not quantify the number and distribution of ipsilaterally projecting RGCs by conventional retrograde DiI labeling from the optic tract to the retina; this method only works reliably from E15.5 onward, when many axons have reached the dorsal thalamus (Godement et al., 1987; Manuel et al., 2008). We therefore analyzed *Nrp1* null mice at E14.5, the latest time point at which mutants were perfectly viable, using a semiquantitative method that measures the relative fluorescence in the ipsilateral optic tract and compares it to the sum of fluorescence intensity in both optic tracts (Figure 2D; adapted from Herrera et al., 2003). This so-called ipsilateral index was increased 5-fold in mutants compared to wild-type littermates (wild-types: 0.08 ± 0.02; mutants: 0.38 ± 0.06; n = 10 each; p < 0.001; Figure 2D). This finding confirms that loss of NRP1 increases the proportion of RGC axons that project ipsilaterally.

### Loss of NRP1 Does Not Perturb the Expression of Midline Markers with a Known Role in Axon Guidance at the Optic Chiasm

A defective midline glial scaffold is in part responsible for the erroneous ipsilateral projection of RGCs in zebrafish *belladona/lhx2* mutants (Seth et al., 2006). We therefore analyzed sections through the optic chiasm of *Nrp1* null mutants with two established markers for midline glia, RC2 and *NrCAM* (Marcus et al., 1995; Williams et al., 2006). However, there were no obvious differences in the arrangement of the RC2-positive glia (Figure 2E), and *NrCAM* was still expressed by these cells (Figure S2B). The CD44/SSEA-positive neurons at the posterior border of the developing optic chiasm, which are required for RGC axon extension across the midline (Marcus et al., 1995; Sretavan et al., 1995), were also present in *Nrp1* null mutants (Figure S2C). Finally, we looked at the expression of the ephrin B2 gene (*Efnb2; ephrin-B2*), which encodes the guidance cue that repels EPHB1-expressing RGC axons from the midline to steer them into the ipsilateral path (Williams et al., 2003). However, ephrin B2 expression at the chiasmatic midline was similar in mutants and wild-types (Figure 2E). We conclude that the architecture of the optic chiasm is not obviously perturbed in *Nrp1* null mutants.

### Loss of NRP1 Does Not Affect Specification of Ipsilateral RGCs

We next asked if the increased ipsilateral projection in *Nrp1* null mutants was due to an enlargement of the retinal domain that gives rise to ipsilaterally projecting RGCs. These neurons arise in two overlapping phases in the mouse. An early but transient ipsilateral projection arises from RGCs in the dorsocentral retina between E12.5 and E14.5; subsequently, RGCs located predominantly in the ventrotemporal retina establish the permanent ipsilateral projection between E14.5 and E16.5 (Godement et al., 1987; Williams et al., 2003, 2006). Consistent with previous studies, *Ephb1* was expressed in the E14.5 wild-type dorsocentral retina, where the RGCs forming the early ipsilateral projection arise (Figure 2F). This expression domain appeared similar in *Nrp1* null mutants (Figure 2F). Due to lethality at E15.5, we were not able to examine *Ephb1* expression in RGCs forming the permanent ipsilateral projection in *Nrp1* null mutants.

ZIC2 is a transcription factor that is both necessary and sufficient to specify the permanent ipsilateral RGCs and is expressed prior to *Ephb1* in these cells and by undifferentiated cells in the ciliary margin (Figure 2F; see Herrera et al., 2003; Tian et al., 2008). Importantly, the *Zic2* expression pattern was similar in *Nrp1* null mutants and controls, with no expansion of the normal expression domain within the RGC layer or ectopic expression by RGCs in other regions of the retina (Figure 2F). We conclude that NRP1 signaling does not regulate chiasm development by affecting the specification of RGCs that give rise to the transient or permanent ipsilateral projections.

### Expression Pattern of Class 3 *SEMA* and *Vegfa* Genes at the Optic Chiasm

**Figure 3:**
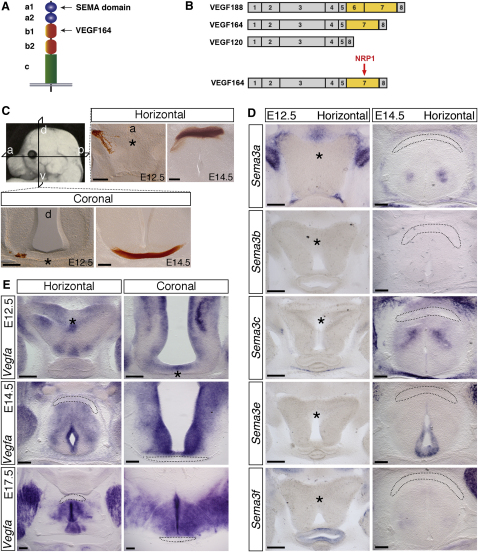
Expression of Class 3 SEMAs and *Vegfa* at the Developing Optic Chiasm (A) Schematic representation of the NRP1 regions that are essential for VEGF164 binding versus binding of the SEMA domain of class 3 SEMAs. (B) Domain structure of the three major mouse VEGF-A isoforms; the exon 7-encoded domain in VEGF164 mediates NRP1 binding. (C) Plane of sections through the optic chiasm and representative images of RGC axons at the chiasmatic midline at E12.5 and E14.5; RGC axons were labeled anterogradely with DiI, and the DiI photoconverted to a brown reaction product. (D and E) In situ hybridization of horizontal sections of wild-type embryos at the level of the optic chiasm with probes specific for *Sema3a–3f* (D) and of horizontal and coronal sections with a probe specific for *Vegfa* (E). Asterisks indicate the position in the E12.5 diencephalon where the optic chiasm will form; dotted lines indicate the position of the optic chiasm at older stages. Horizontal sections: anterior, up; coronal sections: dorsal, up. Scale bars: 200 μm.

We next asked which NRP1 ligand promotes axon crossing at the optic chiasm. There are two types of secreted neuropilin ligands, class 3 SEMAs and VEGF164 (reviewed by Schwarz and Ruhrberg, 2010). Class 3 SEMAs bind the neuropilin a1 domain through their conserved SEMA domain, while VEGF164 binds the b1 domain (Figure 3A). VEGF164 is one of three major VEGF isoforms, named according to the number of amino acids in the mature protein, and binds to NRP1 via an exon 7-encoded domain that is not present in VEGF120 (Figure 3B; Gitay-Goren et al., 1996; Soker et al., 1996, 1998). It is not known if the larger VEGF188 also binds NRP1, because VEGF188 cannot be produced for biochemical studies.

To determine the expression pattern of class 3 SEMAs versus VEGF-A at the optic chiasm, we performed in situ hybridization on sections through the optic chiasm at E12.5 and E14.5 (Figure 3C). We found that none of the five SEMA genes examined were expressed anywhere near the chiasm at E12.5 (Figure 3D). At E14.5, *Sema3b* or *Sema3f* expression was still not detectable anywhere near the chiasm, and the expression domains of *Sema3a*, *Sema3c*, and *Sema3*e in the diencephalon were positioned far posterior to the RGC axon path (Figure 3D).

By contrast, in situ hybridization demonstrated expression of *Vegfa* at the chiasmatic midline (Figure 3E). At E12.5, when the first RGC axons begin to grow into the diencephalon, *Vegfa* was expressed already at the ventral midline, where the chiasm is destined to form (asterisks in Figure 3E). Moreover, expression was strong near the area where RGC axons were extending through the chiasm at E14.5 and was maintained in this area until at least E17.5 (Figure 3E). *Vegfa* is therefore expressed in a pattern that is consistent with a role in RGC axon guidance at the optic chiasm.

### SEMA Signaling through Neuropilins Is Not Essential for RGC Axon Guidance at the Optic Chiasm

**Figure 4:**
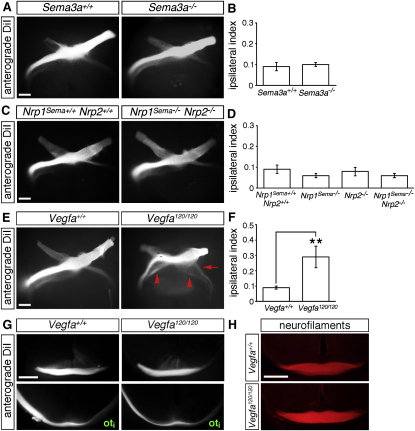
Loss of VEGF164, but Not SEMA Signaling, Impairs RGC Axon Guidance at the Optic Chiasm (A, C, and E) Wholemount views of RGC axons, labeled anterogradely with DiI in E14.5 littermates expressing or lacking *Sema3a* (A), with or without SEMA signaling through neuropilins (*Nrp1^Sema−/−^ Nrp2^−/−^*; C) or expressing or lacking VEGF164 (*Vegfa^120/120^*; E); ventral view, anterior, up. In *Vegfa^120/120^* mutants, both optic tracts are defasciculated; red arrow indicates the normal position of the ipsilateral projection; red arrowheads, the secondary tract and axon defasciculation in the mutants. (B, D, and F) Mean (±SEM) ipsilateral index at E14.5 (*Sema3a^+/+^*, n = 3; *Sema3a^−/−^*, n = 4; *Nrp1^Sema+/+^ Nrp2^+/+^*, n = 5; *Nrp1^Sema−/−^* and *Nrp2^−/−^*, n = 7 each; *Nrp1^Sema−/−^ Nrp2^−/−^*, n = 2; *Vegfa^+/+^* and *Vegfa^120/120^*, n = 14 each); ^∗∗^p < 0.01. (G and H) Coronal sections through the optic chiasm (top panels) and site where the optic tracts begin to diverge (bottom panels), after anterograde DiI labeling (G) or immunolabeling with neurofilament antibodies (H). Scale bars: 250 μm.

Our in situ hybridization studies suggested that the main NRP1-binding SEMA, *Sema3a*, was not expressed at the site where the optic chiasm forms. Because we could not exclude the possibility that SEMA3A diffuses from distant sites of expression into the chiasmatic region, we examined RGC axon guidance in *Sema3a* null mutants (Taniguchi et al., 1997). Anterograde DiI labeling demonstrated that the size and organization of both optic tracts was normal in all four *Sema3a* null mutants examined (Figures 4A and 4B). Together with the expression study, these results establish that NRP1 does not function as a SEMA3A receptor during RGC axon guidance in the mouse.

We next asked whether functional redundancy of SEMA3A with other NRP1-binding class 3 SEMAs, such as those whose expression pattern we had not examined, was responsible for the lack of phenotype in *Sema3a* null mutants. To address this possibility, we took advantage of a mouse mutant that carries point mutations in the a1 domain of NRP1 that abolish the binding of all class 3 SEMAs, but not VEGF164, to NRP1 (*Nrp1^Sema−/−^* mice; Gu et al., 2003; Figure 3A). We found that the size and organization of both optic tracts were normal in all seven *Nrp1^Sema−/−^* mutants examined (Figure 4D).

Finally, to exclude functional compensation for SEMA signaling through NRP1 by NRP2, we examined mice deficient in NRP2 (*Nrp2^−/−^*) or in SEMA signaling through both neuropilins (*Nrp1^Sema−/−^ Nrp2^−/−^* mutants; Gu et al., 2003). The size and organization of both optic tracts was normal in seven out of seven *Nrp2* null and two out of two compound neuropilin mutants (Figures 4C and 4D). We conclude that SEMA signaling through neuropilins is not essential for RGC pathfinding at the mouse optic chiasm.

### Loss of VEGF164 Phenocopies the Chiasm Defect of *Nrp1* Null Mice

Because loss of SEMA signaling cannot explain the optic chiasm defects of *Nrp1* null mice, we asked if the alternative NRP1 ligand VEGF164 regulates RGC pathfinding. To address this possibility, we analyzed *Vegfa^120/120^* mice, which cannot make NRP1-binding VEGF164 or VEGF188, but express VEGF120 to support blood vessel formation (Ruhrberg et al., 2002). Anterograde DiI labeling revealed that 13/14 *Vegfa^120/120^* mutants displayed a range of RGC axon pathfinding errors that were strikingly similar to those caused by loss of NRP1, but were never seen in any of 13 wild-type littermates (Figure 4E). Thus, wholemount preparations showed that both the ipsilateral and contralateral optic tracts were defasciculated in the mutants, with the majority of axons organized into two discrete bundles; consequently, the characteristic asymmetry in the width of the optic tracts was lost (Figure 4E). Moreover, the ipsilateral index was increased significantly in the mutants, suggesting an increase in the proportion of axons that projected ipsilaterally, similar to *Nrp1* null mutants (*Vegfa^+/+^*, 0.09 ± 0.01; versus *Vegfa^120/120^*, 0.29 ± 0.07; p < 0.01; Figure 4F). Coronal sections through DiI-labeled brains (Figure 4G) and neurofilament immunofluorescence staining (Figure 4H) did not reveal additional guidance errors. Based on the striking phenotypic similarities between *Nrp1* and *Vegfa^120/120^* mutants (compare Figures 2A–2D with Figures 4E–4G), we conclude that VEGF164 is the principal NRP1 ligand that promotes RGC axon crossing at the optic chiasm and optic tract organization.

### Loss of VEGF164 Does Not Affect Retinal Organization

Because VEGF-A signaling through FLK1 (KDR/VEGFR2) has been proposed to regulate retinal progenitor cell proliferation and differentiation in the chick (Hashimoto et al., 2006), we examined the expression pattern of VEGF-A and its receptors in the developing eye. *Vegfa* was expressed in the neural retina during the period of RGC development (Figure S3A). Its main vascular VEGF-A receptors, FLT1 (VEGFR1) and FLK1, were expressed by choroidal and hyaloid blood vessels, as expected (Figure S3B, arrowheads). In addition, *Flk1*, but not *Flt1*, was expressed in the neuroblastic layer of the retina (Figure S3B). We therefore examined if a defective retinal architecture contributes to the RGC pathfinding errors in *Vegfa^120/120^* mutants. However, labeling of retinas from E15.5 *Vegfa^120/120^* embryos and wild-type littermates with a marker for mitotic cells (phosphohistone-H^3^) and three different markers for differentiated retinal cells (BRN3A for RGCs; ISL1/2 and PAX6 for RGCs and amacrine cells) did not reveal any obvious defects in retinal organization or lamination (Figure S3C). Thus, mitotic cells were located at the outer surface at the retina, and differentiated neural cells, at the inner surface in a pattern similar to that of wild-types (Figure S3C). The eyes of *Vegfa^120/120^* mutants at E15.5 were smaller than those of wild-type littermates, owing to reduced choroidal vascular growth (Marneros et al., 2005; Saint-Geniez et al., 2006). However, microphthalmia in itself does not cause RGC axon guidance errors at the optic chiasm (Deiner and Sretavan, 1999). Moreover, the thickness of the RGC layer was not obviously different in mutant and wild-type littermates (*Vegfa^+/+^*, 15.2 ± 0.6 μm, n = 3; versus *Vegfa^120/120^*, 15.0 ± 1.0 μm, n = 4), and RGC axons projected normally toward the optic disc and out of the eye in the mutants (Figure S3D). The optic chiasm defects caused by loss of VEGF164 can therefore not be explained by a defective retinal architecture.

### Loss of VEGF164 Promotes the Ipsilateral Projection of RGCs Originating in both the Temporal and Nasal Retina

**Figure 5:**
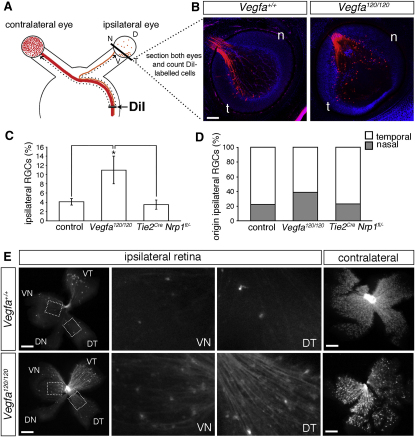
VEGF164 Is Essential for Contralateral Projection at the Optic Chiasm (A) Schematic illustration of the method used to retrogradely label and quantify the relative size of the ipsilateral and contralateral projections. DiI crystals were placed into the dorsal thalamus to label RGC axons in the optic tract on one side of the embryo. After dye diffusion into the ipsilateral and contralateral retinas, eyes were sectioned horizontally to quantify the number of labeled RGCs (B–D) or flatmounted to visualize the distribution of labeled cells within the retina (E). (B) Horizontal sections through the ventral ipsilateral retina in an E15.5 *Vegfa^120/120^* mutant and stage-matched wild-type following retrograde labeling from the optic tract; n, nasal; t, temporal. (C and D) Mean (± SEM) proportion of ipsilateral RGCs relative to total number of RGCs in both eyes (C) and proportion of ipsilateral RGCs originating in temporal versus nasal half of the ipsilateral retina (D) in E15.5 stage-matched wild-types, *Vegfa^120/120^* mutants, and mutants lacking NRP1 in blood vessel endothelium (*Tie2^cre^ Nrp1^fl/–^*); ^∗^ = p < 0.05 compared to wild-type or *Tie2^cre^ Nrp1^fl/–^* conditional mutants (wild-type, n = 8; *Vegfa^120/120^*, n = 6; *Tie2^Cre^ Nrp1^fl/–^*, n = 5). (E) Flatmounted ipsilateral and contralateral retinas from E15.5 *Vegfa^+/+^* and *Vegfa^120/120^* embryos after retrograde labeling from the optic tract. The boxed regions are shown at higher magnification in the adjacent panels. DT, dorsotemporal; VN, ventronasal; DN, dorsonasal; VT, ventrotemporal. Scale bars: 125 μm.

Because *Vegfa^120/120^* embryos survive to birth, we confirmed the increase in the ipsilateral projection by counting all DiI-labeled cells in sections through the entire ipsilateral and contralateral eye after retrograde labeling from the optic tract (Figure 5A). This demonstrated a significant increase in the proportion of DiI-labeled cells in the ipsilateral retina of E15.5 *Vegfa^120/120^* mutants relative to stage-matched wild-types (wild-type, 4.2% ± 0.7%, n = 8; *Vegfa^120/120^*, 11.1% ± 3.0%, n = 6; p < 0.05; Figures 5B and 5C). The spatial origin of the ipsilaterally projecting cells was also altered. In wild-types, most ipsilateral RGCs were restricted to the ventrotemporal region of the retina as expected (Figure 5B). In contrast, many ipsilateral RGCs were located throughout the temporal and nasal retina in the absence of VEGF164 (Figure 5B; wild-types: temporal, 30.8 ± 10.5, nasal, 7.8 ± 5.5; *Vegfa^120/120^*: temporal, 85.3 ± 24.3, nasal, 48.8 ± 21.1).

We next determined the proportion of ipsilaterally projecting RGCs in the nasal retina relative to the temporal retina. As expected, most ipsilaterally projecting RGCs originated in the temporal retina of wild-types (temporal, 78.3% ± 2.5%, versus nasal, 21.7% ± 2.5%; Figures 5B and 5D). Consistent with the normal specification of the *Zic2*-positive domain in the ventrotemporal retina in mutants lacking the VEGF164 receptor NRP1 (Figure 2F), the majority of ipsilaterally projecting RGCs also originated in the temporal retina when VEGF164 signaling was lost (61.1% ± 4.2%; Figure 5D). However, the proportion of ipsilaterally projecting RGCs located in the nasal retina was increased almost 2-fold compared with that of stage-matched wild-type controls (wild-type nasal retina, 21.7% ± 2.5%, versus mutant nasal retina, 38.9% ± 4.2%; p < 0.05; Figure 5D). Flatmounted retinas confirmed that a greater proportion of axons projected ipsilaterally in *Vegfa^120/120^* mutants compared with wild-types, and that the excess ipsilaterally projecting neurons originated throughout the retina (Figure 5E). Conversely, fewer neurons were labeled in the contralateral retina of mutants compared with wild-types (Figure 5E). Loss of VEGF164 therefore increases the number of ipsilaterally projecting RGC axons at the expense of contralaterally projecting RGCs.

### Loss of NRP1 in Blood Vessels Does Not Affect Midline Crossing of RGC Axons

Because VEGF164 signals through NRP1 in blood vessels and because NRP1 organizes blood vessels in the brain (Soker et al., 1998; Gerhardt et al., 2004), we asked if defective blood vessel pattering was responsible for impaired axon crossing at the optic chiasm in *Vegfa^120/120^* and *Nrp1* null mutants by counting all retrogradely labeled RGCs in sections through the entire ipsilateral and contralateral eyes of embryos lacking NRP1 specifically in blood vessels (*Tie2^Cre^ Nrp1^fl^*^/−^; Gu et al., 2003). In contrast to the *Vegfa^120/120^* mutants, the vessel-specific *Nrp1* mutants contained a normal proportion of ipsilaterally projecting RGCs (3.6% ± 1.0%, n = 5; Figure 5C). Moreover, the cell bodies of ipsilaterally projecting RGCs were distributed normally within the retina, with the vast majority being derived from the temporal retina (77.0% ± 4.8%, n = 5; Figure 5D). Because endothelial-specific *Nrp1* null mutants display microphthalmia and vascular brain abnormalities similar to those of full *Nrp1* null and *Vegfa^120/120^* mutants (Gu et al., 2003; Fantin et al., 2010), reduced eye size or defective blood vessel patterning cannot explain the decreased midline crossing of RGC axons in the absence of VEGF164/NRP1 signaling. We conclude that VEGF164/NRP1 signaling promotes contralateral axon crossing at the chiasmatic midline independently of blood vessels.

### VEGF164 Promotes RGC Axon Extension

**Figure 6:**
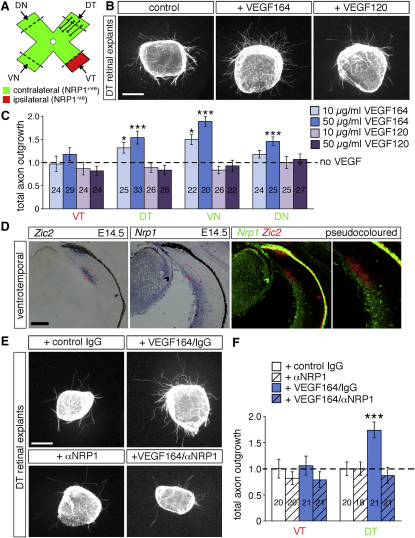
VEGF164, but Not VEGF120, Promotes Outgrowth of Contralateral RGCs (A) Schematic illustration of the retinal areas placed into culture. Explants from peripheral ventrotemporal (VT) retina contain predominately ipsilaterally projecting RGCs, whereas peripheral dorsotemporal (DT), ventronasal (VN), and dorsonasal (DN) explants contain mainly contralaterally projecting RGCs. (B) Retinal explants from E14.5 wild-type dorsotemporal retina cultured for 24 hr in collagen gels in control culture medium or medium containing VEGF164 (50 ng/ml) or VEGF120 (50 ng/ml), fixed and stained for β-tubulin. (C) Mean (±SEM) total axon outgrowth from explants cultured in the presence of VEGF164 or VEGF120 (10 or 50 ng/ml), normalized to outgrowth in control cultures containing no exogenous VEGF (indicated with a dashed line). Number of control explants, 27–29 per quadrant; number of explants cultured with VEGF is indicated on the bars. ^∗^p < 0.05, ^∗∗∗^p < 0.001 compared to controls. (D) In situ hybridization with probes specific for *Zic2* or *Nrp1* on adjacent 20 μm sections through the E15.5 ventrotemporal wild-type retina. Images in right-hand panels were pseudocolored and overlaid to demonstrate the mutually exclusive expression pattern of both genes. (E) Retinal explants from E14.5 wild-type dorsotemporal retina cultured for 24 hr in medium containing or lacking VEGF164 (50 ng/ml) plus control goat IgG (1 μg/ml) or αNRP1 (0.5 μg/ml) and immunolabeled for β-tubulin. (F) Mean (±SEM) total axon outgrowth from explants cultured in the presence of control IgG (1 μg/ml) or αNRP1 (0.5 μg/ml) in the presence or absence of VEGF164 (50 ng/ml), normalized to the outgrowth in cultures containing control IgG alone (indicated with a dashed line). The number of explants per condition is indicated on the bars. ^∗∗∗^p < 0.001 compared to control IgG. Scale bar, 200 μm.

The expression pattern of VEGF-A in the diencephalon raised the possibility that it promotes the growth of NRP1-expressing RGC axons at the chiasmatic midline. To test this hypothesis, we explanted the peripheral region of all four quadrants of E14.5 retinas (Figure 6A) and assayed the response of RGC axons to recombinant VEGF-A on collagen or laminin (Figures 6B, 6C, S4A, and S4B). On both substrates, VEGF164 significantly increased outgrowth in a dose-dependent manner from the retinal regions that give rise to contralaterally projecting RGCs (dorsotemporal, ventronasal, dorsonasal; Figures 6B, 6C, S4A, and S4B). In contrast, outgrowth from the ventrotemporal retina, the origin of ipsilaterally projecting RGCs, was not altered significantly (Figures 6C and S4B). Addition of VEGF120 did not promote axon outgrowth from any retinal region (Figures 6B, 6C, S4A, and S4B).

Consistent with the failure to respond to VEGF164, *Nrp1* was not expressed at detectable levels in the *Zic2*-positive ventrotemporal crescent that gives rise to ipsilateral RGCs; in contrast, *Nrp1* was expressed in RGCs outside the *Zic2* domain (Figure 6D). The mutually exclusive expression pattern of *Nrp1* and *Zic2* was particularly evident when adjacent sections for both markers were pseudocolored and overlaid. This observation suggests that VEGF164 promotes axon outgrowth only from RGCs that express NRP1.

To confirm that VEGF164 promotes RGC axon growth in a NRP1-dependent fashion, we used a function-blocking antibody specific for NRP1 (Fantin et al., 2010). Control experiments demonstrated that axon outgrowth in the absence of VEGF164 was not altered by isotype control IgG or NRP1 antibody and that outgrowth from ventrotemporal retina, where RGCs lack NRP1 expression, remained at baseline levels when VEGF164 was added together with control IgG or NRP1 antibody (Figures 6E and 6F). In contrast, axon outgrowth from NRP1-positive dorsotemporal explants was increased significantly when VEGF164 was added together with IgG and this VEGF164-induced enhancement of growth was blocked completely by the NRP1 antibody (Figures 6E and 6F). We conclude that VEGF164 promotes the growth of presumptive contralaterally projecting RGC axons through its receptor, NRP1.

Previous studies demonstrated a role for the NRP1 coreceptor FLK1 in axon regeneration after VEGF treatment of postnatal RGC explants (Böcker-Meffert et al., 2002). However, *Flk1* was not expressed obviously in RGCs at E12.5 or E14.5, when they extend axons through the chiasm (Figure S3B). Consistent with this finding, a previously validated function-blocking antibody that is specific for FLK1 and blocks VEGF-A signaling in endothelial cells (Gerhardt et al., 2003) did not inhibit the response of RGC axons to VEGF164 (Figures S4C and S4D). We conclude that VEGF164 signals through NRP1 in embryonic RGC axons independently of FLK1.

### VEGF164 Is a Chemoattractant for RGC Axon Growth Cones

**Figure 7:**
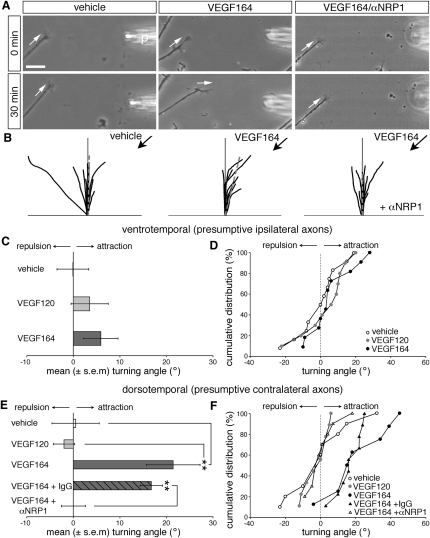
VEGF164 Is a Chemoattractant for RGC Growth Cones (A) RGC growth cones at 0 min and 30 min after exposure to a gradient of vehicle (PBS), VEGF164, or VEGF164 in the presence αNRP1; the gradient emanated from a pipette (p), placed at a distance of 100 μm and a 45° angle relative to the growth cone; white arrows indicate the direction of growth cone extension. (B) Superimposed RGC axon trajectories over the 30 min observation period; black arrows indicate the direction of the gradient. (C and D) Mean (±SEM) turning angle (C) and cumulative frequency curves (D) of RGC growth cones from the ventrotemporal retina. The turning induced by VEGF120 or VEGF164 was not significantly different from the turning induced by PBS (C). For cumulative frequency curves, the turning angle of each growth cone was plotted against the percentage of growth cones turning to that angle or less. (E and F) Mean (±SEM) turning angle (E) and cumulative frequency curves (F) of RGC growth cones from the dorsotemporal retina. VEGF164 induced significant attraction relative to PBS or VEGF120 (^∗∗^p < 0.01); the response was abrogated by αNRP1, but not control IgG. Scale bar: 25 μm.

To address if VEGF acts directly on RGC axons as a guidance signal, we used the growth cone turning assay (Lohof et al., 1992). In this assay, a pipette is placed at an angle of 45° to the initial direction of axon extension, and test substances are puffed into the medium to establish a gradient. As expected, we found that growth cones from both ventrotemporal retina, which gives rise to NRP1-negative, ipsilaterally projecting RGCs, and dorsotemporal retina, which gives rise to NRP1-positive, contralaterally projecting RGCs, grew randomly in a gradient of PBS (Figures 7A–7F; mean turning angle of ventrotemporal axons: −0.1° ± 3.4°, n = 12; mean turning angle of dorsotemporal axons: 0.5° ± 5.1°, n = 10). Random growth of both ventrotemporal and dorsotemporal growth cones occurred also in a VEGF120 gradient (Figures 7C–7F and S5; mean turning angle of ventrotemporal growth cones: 3.5° ± 4.0°, n = 10; mean turning angle of dorsotemporal growth cones: −2.0° ± 2.3°, n = 9). We also found that VEGF164 did not induce significant turning of ventrotemporal growth cones (Figures 7C and 7D; mean turning angle: 5.9° ± 3.7°, n = 11). In contrast, dorsotemporal RGC growth cones were attracted strongly by a gradient of VEGF164 (Figures 7A, 7B, 7E, and 7F; mean turning angle: 21.5° ± 5.8°, n = 9, p < 0.01 compared to PBS). This attractive turning response was abrogated effectively by the function-blocking NRP1 antibody, whereas control IgG had no effect (Figures 7A, 7B, 7E, and 7F). The mean turning angle evoked by VEGF164 in the presence of control IgG was 16.8° ± 2.4° (n = 9), but 0.0° ± 2.6° (n = 10) in the presence of the function-blocking anti-NRP1 antibody (p < 0.001). VEGF164 therefore signals through NRP1 to attract the growth cones of presumptive contralateral RGC axons.

Based on these findings, together with the expression pattern of VEGF164 and NRP1 and the loss-of-function phenotypes of the corresponding mouse mutants in vivo, we conclude that VEGF164 signals to NRP1-expressing RGC growth cones to promote axon crossing at the chiasmatic midline.

## Discussion

**Figure 8:**
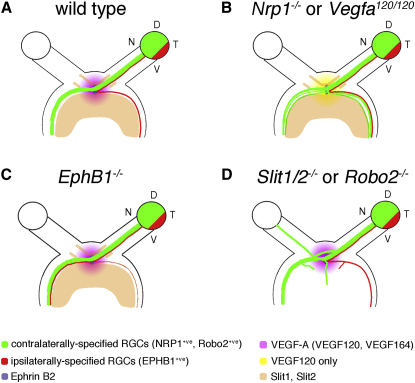
Working Model for Axon Guidance at the Developing Mouse Optic Chiasm (A) In wild-type mice, VEGF164 at the chiasmatic midline counteracts inhibitory cues to promote the contralateral growth of NRP1-expressing axons, while repulsive ephrin B2 signals to EPHB1-expressing, NRP1-deficient axons to promote ipsilateral projection. Repulsive SLIT1 and SLIT2 signals cooperate to narrow the VEGF164-positive corridor through which RGC axons travel. (B) In the absence of VEGF164 signaling through NRP1, some RGC axons destined for the contralateral tract cannot overcome the inhibitory midline environment and form ectopic ipsilateral projections; in addition, the optic tracts defasciculate. (C) In the absence of ephrin B2 signaling through EPHB1, ipsilateral axons are no longer repelled from the midline and project contralaterally. (D) In the absence of SLIT signaling through Robo receptors, RGC axons are not constrained to the normal optic path and cross the VEGF164-positive midline region in a broader domain.

Nerves and blood vessels ramify through tissues in strikingly similar patterns and develop during embryogenesis under the control of similar cellular and molecular mechanisms (reviewed by Ruiz de Almodovar et al., 2009 and Adams and Eichmann, 2010). Thus, classical axon guidance cues of the ephrin, netrin, and SLIT families affect the growth of blood vessels. Conversely, it has been hypothesized that the main vascular growth factor VEGF-A is important for axon growth and guidance, either in its own right or by competing with SEMA3A for NRP1 binding (reviewed by Carmeliet, 2003 and Ruiz de Almodovar et al., 2009). However, evidence is still lacking that VEGF-A controls axon guidance in vivo. By demonstrating that VEGF164 is expressed at the optic chiasm midline, is essential for RGC axon guidance and fasciculation in vivo, and promotes RGC axon outgrowth and attractive growth cone turning, we provide evidence that VEGF-A is a physiological axon guidance cue (Figures 8A and 8B).

### VEGF164 Signals Directly to RGC Axons to Promote Contralateral Axon Growth

We found that loss of VEGF164 or its receptor, NRP1, perturbs axon crossing at the optic chiasm in a similar manner in vivo, causing optic tract defasciculation and increasing ipsilateral projection. Because VEGF and NRP1 are well known for their essential roles in blood vessel growth (Kawasaki et al., 1999; Ruhrberg et al., 2002; Gerhardt et al., 2004), we used endothelial-specific NRP1 mutants to exclude the possibility that loss of VEGF164 signaling inhibits contralateral axon growth indirectly by disrupting the brain vasculature. These mutants suffer blood vessel defects similar to those seen in full NRP1 knockouts (Gu et al., 2003), but do not display defects in midline crossing of contralateral RGC axons. VEGF164/NRP1 signaling therefore controls axon crossing at the optic chiasm independently of its role in blood vessels. Instead, our results support a model in which VEGF164 signals through NRP1 in RGC growth cones to regulate axon pathfinding directly (Figure 8B). Thus, we found that NRP1 is expressed strongly by contralateral RGC axons throughout the period of optic chiasm development, and that VEGF164 is a powerful chemoattractant for growth cones from presumptive contralateral RGC axons that acts in a NRP1-dependent fashion. In contrast, the pan-VEGF isoform receptor FLK1 was not expressed in developing RGCs and was not required for the growth-promoting effect of VEGF164. Moreover, the FLK1-binding VEGF120 isoform did not promote axon growth or growth cone turning in vitro. These findings suggest that NRP1 controls the behavior of developing RGC axons independently of its vascular coreceptor FLK1, or indeed FLT1, which also is not expressed by developing RGCs. Future studies might therefore examine if NRP1 in RGC axons signals through its cytoplasmic tail or recruits a coreceptor that is not a classical VEGF receptor.

### VEGF164 Acts Independently of Class 3 SEMAs to Guide Contralateral Axons

VEGF164 has been hypothesized to regulate axon guidance based on its ability to compete with SEMA3A for NRP1 binding (Carmeliet, 2003). However, we could not identify an essential role for SEMA signaling through NRP1 in optic chiasm development in mice. Accordingly, neither the genetic ablation of SEMA3A, nor the loss of SEMA signaling through NRP1 alone or both neuropilins together, perturbed optic chiasm development. These findings were surprising, because the NRP1 ligand SEMA3D provides repulsive signals that channel RGC axons into the contralateral optic tract in zebrafish (Seth et al., 2006). A possible explanation for the class 3 SEMA requirement in fish, but not mammals, is that fish have an exclusive contralateral projection. It will therefore be interesting to investigate whether VEGF-A signaling at the chiasm midline is conserved in all vertebrates, independently of SEMAs, or if there is a species-dependent specialization with respect to the choice of NRP1 ligand. Interestingly, even *Drosophila*, a species without a circulatory system, has a VEGF-A homolog that promotes cell migration (Traver and Zon, 2002). This raises the possibility that VEGF-A plays evolutionary conserved roles in the nervous system that predates its function in blood vessels.

### VEGF164 Is an Attractive Midline Cue for Commissural Axons at the Optic Chiasm

Previous in vitro experiments raised the possibility that a growth-promoting factor for commissural axons is present at the chiasm midline (Tian et al., 2008). However, the molecular identity of this factor has never been established. The only molecule found previously to promote contralateral RGC axon growth is the cell adhesion molecule NrCAM. However, NrCAM is not the elusive midline cue that promotes commissural axon crossing at the optic chiasm, because it acts as a receptor within RGC axons rather than as a guidance signal at the chiasm midline (Williams et al., 2006). In the vertebrate spinal cord, commissural axons are attracted to the midline by the combined action of the chemoattractants netrin 1 and SHH (Serafini et al., 1996; Charron et al., 2003). However, neither of these molecules is expressed at the chiasm midline or promotes contralateral RGC axon extension (Deiner and Sretavan, 1999; Marcus et al., 1999; Trousse et al., 2001; Sánchez-Camacho and Bovolenta, 2008). In contrast, VEGF-A is expressed strongly at the chiasm midline, is required for normal contralateral projection, and is growth promoting and chemoattractive for RGC axons. We therefore propose that VEGF-A is a positive signal for RGC axons and one of the long-sought-after midline factors that promotes commissural axon crossing at the optic chiasm. Because VEGF is expressed in a broad domain around the chiasm, the VEGF164-mediated promotion of RGC growth must be balanced by repulsive cues that refine the area of axon crossing. Consistent with this idea, the chemorepellents SLIT1 and SLIT2 define the boundaries of the corridor through which RGC axons migrate at the chiasm midline, and loss of these repellents causes RGC axons to cross the midline in an abnormally broad domain (Erskine et al., 2000; Plump et al., 2002; Figure 8D).

### VEGF-A Acts Independently of NrCAM to Promote Contralateral Axon Growth

NrCAM modulates neuropilin signaling in response to class 3 SEMAs during commissural axon guidance in the anterior commissure (Falk et al., 2005) and spinal cord (Nawabi et al., 2010). Several lines of evidence argue against the possibility that NrCAM modulates neuropilin signaling in response to VEGF164 at the optic chiasm. First, the chiasm defects of mice lacking NrCAM (Williams et al., 2006; data not shown) versus VEGF164 and NRP1 appear distinct. Second, the temporal requirement for NrCAM versus VEGF164 and NRP1 in contralateral RGC axon guidance differs: defective midline crossing occurs in *Nrp1* null and *Vegfa^120/120^* mutants already at E14.0, when the first RGC axons extend through the chiasm (Godement et al., 1987), while midline crossing in *NrCAM* null mutants is affected only late in development, from E17.5 onward (Williams et al., 2006). Finally, the retinal origin of the excess ipsilateral projections differs, as VEGF164 signaling through NRP1 promotes the contralateral projection of RGCs originating throughout the retina, whereas NrCAM is essential for contralateral growth of a small subset of axons that originate exclusively in the ventrotemporal retina (Williams et al., 2006). Based on these differences, we conclude that NRP1 and NrCAM function independently of each other to promote contralateral axon growth of RGC axons.

### Role for VEGF164/NRP1 Signaling in Optic Tract Fasciculation

In addition to promoting contralateral guidance of RGC axons, we found that VEGF164/NRP1 signaling promotes axon cohesion within the optic tracts. Thus, mutants lacking VEGF164 or NRP1 showed defasciculation of both the ipsilateral and contralateral tract. It is not known if VEGF164 acts as an extrinsic signal in the axonal environment to control fasciculation or, because it is also expressed by RGCs themselves, in a local autocrine fashion. Further in vivo studies, for example with tissue-specific NRP1 knockouts, will be necessary to fully understand this aspect of the phenotype. Interestingly, loss of Dicer, a protein essential for the maturation of regulatory micro RNAs that regulate *Nrp1* among several other targets (Zhou et al., 2008), leads to similar defasciculation and also increases the ipsilateral projection (Pinter and Hindges, 2010).

### Integration of Positive VEGF Signaling with Inhibitory Pathways at the Optic Chiasm

An exquisite balance of attractive and inhibitory cues governs axon crossing at the CNS midline. Explant assays have shown that the spinal cord floor plate is strongly chemoattractive and growth promoting for commissural axons (Tessier-Lavigne et al., 1988; Serafini et al., 1996). There, axons loose responsiveness to midline attractants only upon crossing, and instead become sensitive to repellents such as SLITs that drive them out off the midline territory (Shirasaki et al., 1998; Sabatier et al., 2004). In contrast, explanted chiasm tissue inhibits axon growth (Wang et al., 1995, 1996), and growth cones therefore slow down as they approach this region (Godement et al., 1994; Mason and Wang, 1997). Furthermore, there is no evidence to date that RGC axons acquire responsiveness to repellents as they encounter the midline territory; for example, they are sensitive to inhibitory SLIT signaling both before and after crossing (Thompson et al., 2006a, 2006b). Despite these differences, most RGC axons eventually cross to form the contralateral projection, suggesting that growth-promoting factors exist to help them cross.

We found that in vitro, in the absence of inhibitory chiasm-derived cues, VEGF164 is a powerful growth promoter and chemoattractant for RGC axons. In vivo, VEGF164 also promotes axon crossing, but is not essential for the crossing of all RGCs, presumably because it acts redundantly with other attractive cues to ensure that RGCs overcome the inhibitory chiasm environment. In support of this idea, presumptive ipsilateral RGC axons project contralaterally in the absence of ephrin B2 signaling (Williams et al., 2003), even though they do not normally express NRP1. An essential role for VEGF164 in balancing inhibitory signals at the chiasm midline would also explain why growth cones do not stall at the midline. Thus, inhibitory cues are essential to prevent the trapping of NRP1-expressing RGC axons at the VEGF164-expressing midline and help drive advancing axons into the optic tracts. Additionally, crossed axons may lose sensitivity to VEGF164, because they downregulate an unidentified NRP1 coreceptor or because they upregulate a receptor that increases sensitivity to inhibitory signals after crossing. Identifying further guidance pathways and generating compound mouse mutants will help decide between these possibilities.

### Conclusions

We have identified an attractive and growth-promoting midline signal that overcomes the repulsive environment of the chiasm midline to promote commissural axon growth. This attractive factor is the NRP1-binding VEGF164 isoform of the classical vascular growth factor VEGF-A. While there are many examples of axon guidance signals playing a prominent role in the developing vasculature, physiological evidence for an involvement of angiogenic factors in axon pathfinding was previously lacking. Our findings provide in vivo evidence that VEGF-A is essential for axon pathfinding. Attractive VEGF164/NRP1 signaling in contralaterally projecting RGCs and repulsive ephrin B2/EPHB1 signaling in ipsilaterally projecting RGCs therefore cooperate to sort axons at the optic chiasm into the appropriate tract (Figure 8). Because VEGF is also expressed at the midline in other parts of the nervous system, including the hindbrain and spinal cord (Ruhrberg et al., 2002; Schwarz et al., 2004; Q.S. and C.R., unpublished data), our results may be of general significance for our understanding of the molecular mechanisms that regulate the formation of commissures.

## Experimental Procedures

### Mouse Strains

We used the following mouse strains: *Nrp1* null, *Nrp2* null, *Nrp1^Sema−/−^, Nrp1^fl/fl^*, *Tie2^Cre^*, *Sema3a* null, *Vegfa^120/120^*, *Flt1^LacZ^*, and *Flk1^LacZ^* (Schwarz et al., 2004 and Supplemental Experimental Procedures). All animal procedures were performed in accordance with institutional and UK Home Office guidelines.

### In Situ Hybridization

In situ hybridization was performed as described (Thompson et al., 2006a) with digoxigenin-labeled riboprobes for *Nrp1*, *Nrp2*, *Sema3a–f*, *Vegf164*, *Ephb1, Efnb2*, *Zic2*, *NrCAM, Flk1*, and *Flt1* (Schwarz et al., 2004; Herrera et al., 2003; Williams et al., 2003, 2006; see Supplemental Experimental Procedures).

### Immunofluorescence

Immunostaining was performed as described (Erskine et al., 2000; Thompson et al., 2006b) with antibodies specific for SSEA1, RC2, ISL1/2, or PAX6 (Developmental Studies Hybridoma Bank); phosphohistone-H^3^, BRN3A, or neurofilaments (Millipore); NRP1 (R&D systems); or biotinylated IB4 (Sigma).

### Anterograde and Retrograde DiI Labeling

Anterograde DiI labeling was performed as described (Plump et al., 2002; Thompson et al., 2006a; Figure S2A). NIH Image was used to measure the fluorescent intensity of the ipsilateral and contralateral optic tracts in nonsaturated wholemount images (Figure 2D). Retrograde DiI labeling from the dorsal thalamus was performed as described previously (Manuel et al., 2008; Figure 5A).

### RGC Explant Cultures

Peripheral retina from E14.5 C57 BL/6J was explanted into a 1:1 mixture of bovine dermis and rat tail collagen (BD Biosciences) or onto glass-bottomed dishes (MatTek Corporation) coated with poly-ornithine (Sigma-Aldrich) and 10 μg/ml laminin (Invitrogen), as described (Erskine et al., 2000; Williams et al., 2003). VEGF164 or VEGF120 was added to the culture medium composed of DMEM:F12 (Invitrogen), 1% BSA, and ITS supplement (Sigma-Aldrich). In some experiments, we added 0.5 μg/ml function-blocking goat anti-rat NRP1, 0.3 μg/ml function-blocking goat anti-rat FLK1/VEGFR2 antibody, or 1 μg/ml goat IgG (R&D systems). After 24 hr, the cultures were fixed and stained for β-tubulin (1:500; Sigma). Image J was used to quantify total axon outgrowth. Statistical comparisons were made using ANOVA or the Mann-Whitney U test.

### Growth Cone Turning Assay

Growth cone turning assays were performed using an adaptation of the method of Murray and Shewan (2008). Growth cones were positioned at a 45° angle and 100 μm from a micropipette containing PBS, VEGF164 (50 μg/ml), or VEGF120 (50 μg/ml), and were imaged for 30 min in reagent gradients generated with a Picospritzer III (Intracel). In some experiments, 0.5 μg/ml function-blocking goat anti-rat NRP1 antibody or control IgG was added. The angle turned by the growth cone was calculated using Image J. Statistical comparisons were made using a Mann-Whitney U test.

## Supplemental Information

Document S1. Supplemental Figures and Experimental Procedures
