# Supplementary material for: VEGF Signaling through Neuropilin 1 Guides Commissural Axon Crossing at the Optic Chiasm
Source: Neuron. 2011 Jun 9;70(5):951–65. doi: 10.1016/j.neuron.2011.02.052 (PMC3114076; doi:10.1016/j.neuron.2011.02.052)
Supplement: Document S1. Supplemental Figures and Experimental Procedures [file mmc1.pdf]

Neuron, volume *70*

Supplemental Information

VEGF Signaling through Neuropilin 1 Guides Commissural Axon Crossing at the Optic Chiasm

Lynda Erskine, Susan Reijntjes, Thomas Pratt, Laura Denti, Quenten Schwarz, Joaquim M. Vieira, Bennett Alakakone, Derryck Shewan, and Christiana Ruhrberg

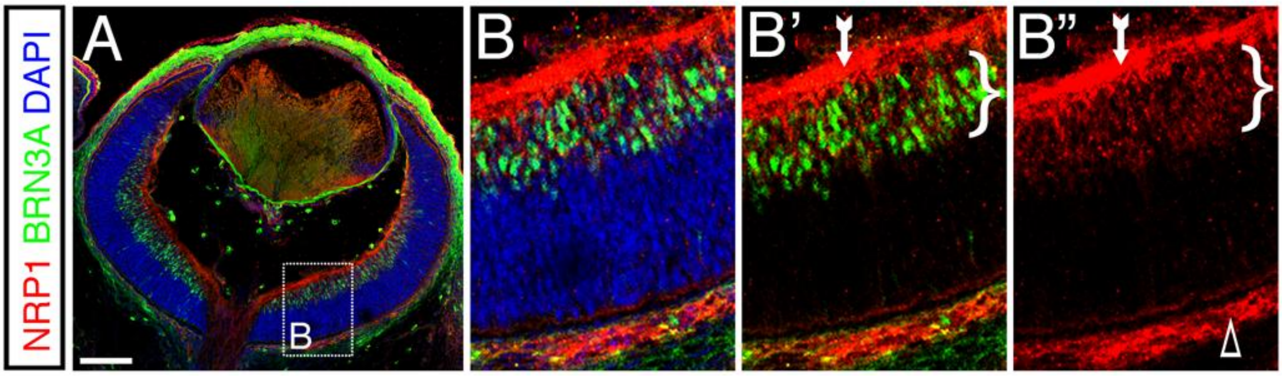

Supplemental Figure 1, related to Figure 1. NRP1 is expressed on mouse RGC axons.
(A) Coronal section of an E14.5 wild type retina, stained by double immunofluorescence with antibodies specific for NRP1 (red) and the RGC marker BRN3A (green); the section was counterstained with the nuclear marker DAPI (blue).
(B) Higher magnification of the boxed region in (A); red and green channels are shown in (B'), the red channel only is shown in (B''). NRP1-positive RGC axons are indicated with feathered arrows, choroidal vessels with clear arrowheads. White bracket indicates the RGC layer.
Scale bar: 100 µm (A).

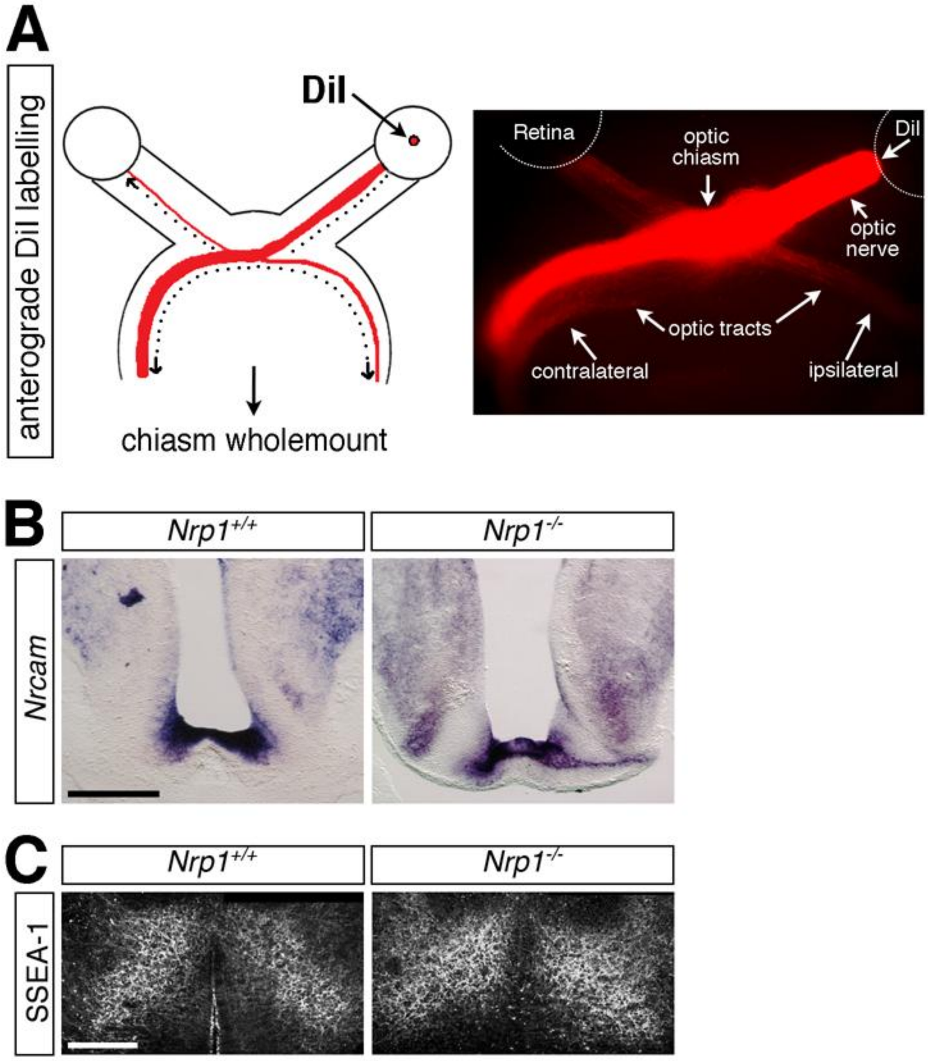

Supplemental Figure 2, related to Figure 2. Anterograde labelling technique and midline architecture in *Nrp1*-null mutants.

(A) Illustration of the anterograde DiI labelling technique, which was used to visualise the projection of RGC axons. A crystal of DiI was placed on the optic disc of one retina. After the dye had diffused along the axons, the brain was dissected and imaged, ventral side up, with a fluorescent stereomicroscope to reveal RGC axons in the optic nerve, chiasm and proximal optic tract, as shown in the adjacent panel.

(B-C) Expression of both *Nrcam* and SSEA-1 is maintained in the absence of *Nrp1*. Coronal (B) and horizontal (C) sections of E14.5 *Nrp1*$^{+/+}$ and *Nrp1*$^{-/-}$ littermates subjected to in situ hybridisation with a probe specific for *Nrcam* (B) to visualise midline radial glia or immunofluorescently labelled to visualise SSEA/CD44-positive neurons, which are located posterior to the optic chiasm and important for axon growth across the midline (C).

Scale bars: 150 µm (B), 200 µm (C).

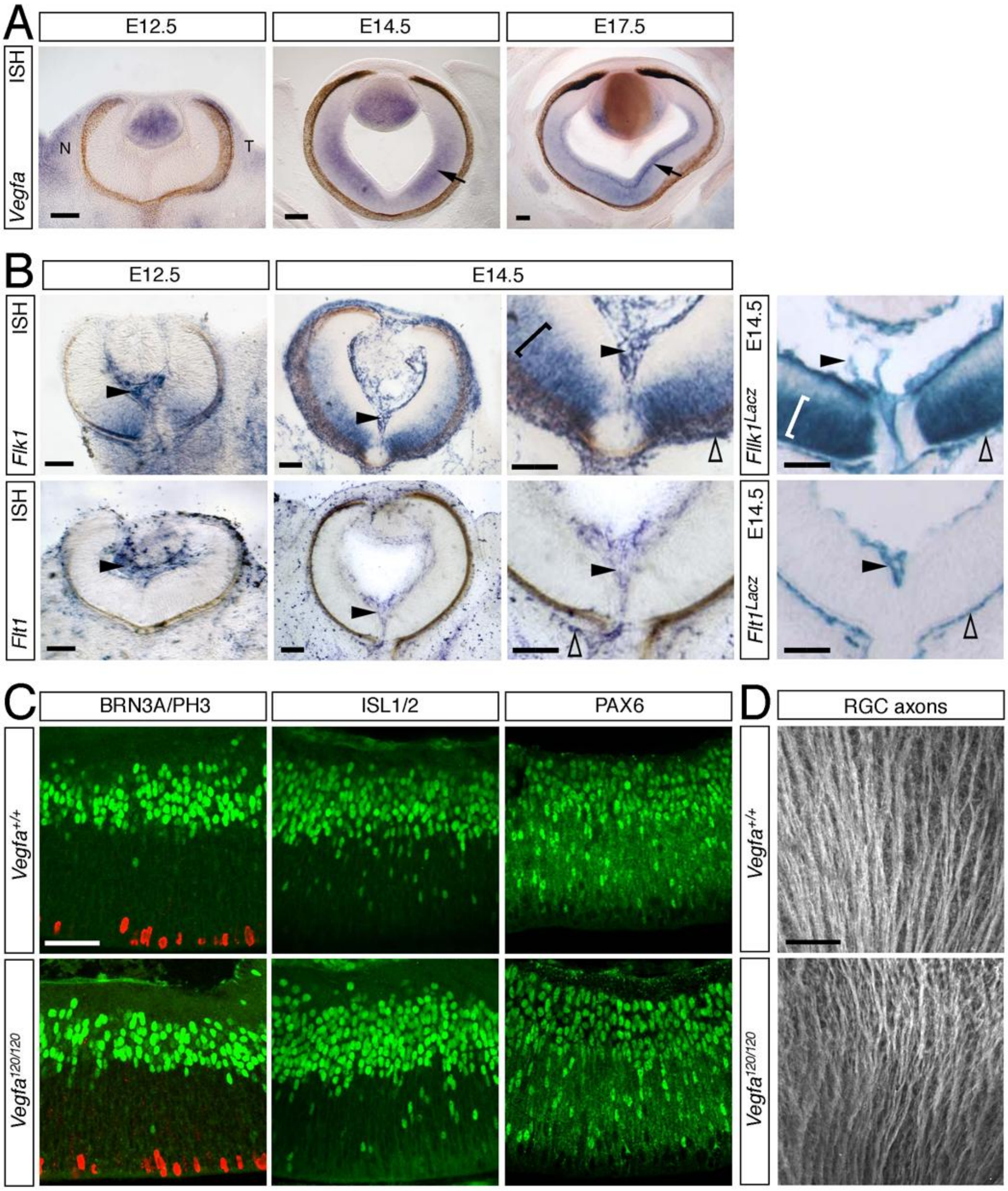

Supplemental Figure 3, related to Figure 4. Retinal organisation is normal in the absence of VEGF164.

(A) In situ hybridisation (ISH) of horizontal sections through E12.5 - E17.5 wild type eyes with probe specific for *Vegfa*; staining in the retina is indicated with arrows; N, nasal; T, temporal.

(B) ISH of horizontal sections through E12.5 and E14.5 wild types with probes specific for *Flt1/Vegfr1* or *Flk1/Vegfr2* and histochemical detection of beta-galactosidase activity in horizontal sections of E14.5 eyes from *Flt1/Vegfr1*^{LacZ} or *Flk1/Vegfr2*^{LacZ} *knock-in* reporter mice. Solid arrowheads indicate hyaloid vessels, clear arrowheads choroidal vessels; brackets indicate the neuroblastic layer.

(C) Immunofluorescence staining of coronal sections of E15.5 *Vegfa*$^{+/+}$ and *Vegfa*$^{120/120}$ retinas with antibodies for phosphohistone H^3 (PH3) to detect mitotic cells (red) in the neuroblastic layer and the RGC marker BRN3A (green), or with antibodies for the RGCs/amacrine cell markers ISL1/2 and PAX6 (green).

(D) Neurofilament stains of retina flatmounts from E14.5 littermate wild types and *Vegfa*$^{120/120}$ mutants.

Scale bars: 100 µm (A, B), 50 µm (C, D).

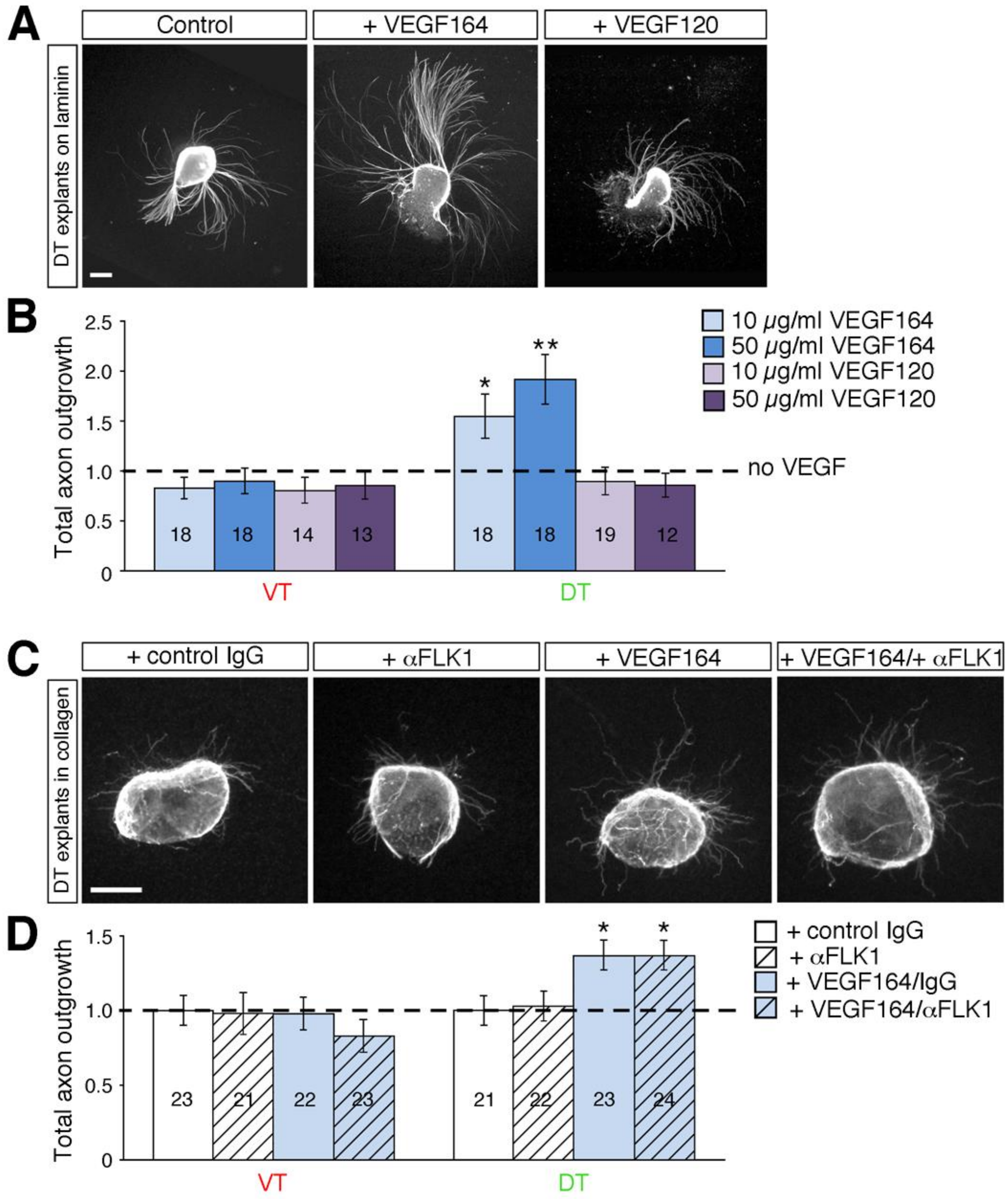

Supplemental Figure 4, related to Figure 6. VEGF164 promotes RGC axon outgrowth independently of FLK1/VEGFR2.

(A) Explants of E14.5 dorsotemporal wild type retina cultured for 24 h on laminin in control culture medium or in medium containing 10 ng/ml or 50ng/ml of VEGF164 or VEGF120 and then fixed and stained with an antibody for β-tubulin.

(B) Mean (± s.e.m.) total axon outgrowth from explants cultured in the presence of 10 or 50 ng/ml VEGF120 or VEGF164, normalised to outgrowth in control cultures containing no exogenous VEGF (indicated with a dashed line). Number of control explants, 19-21 per quadrant; number of explants cultured with VEGF is indicated on the bars. * = $p < 0.05$; ** = $p < 0.01$ compared to controls.

(C) Retinal explants of E14.5 wild type dorsotemporal retina cultured for 24 hrs in control medium or medium containing 10 ng/ml VEGF164 plus control goat IgG (1 μg/ml) or αFLK1/VEGFR2/KDR (0.3 μg/ml) and then stained with an antibody for β-tubulin.

(D) Mean (± s.e.m) total axon outgrowth from explants cultured in goat IgG (1 μg/ml) or αFlk1 (0.3 μg/ml) in the presence or absence of 10 ng/ml VEGF164, normalised to the outgrowth in control cultures containing no exogenous VEGF164 (indicated with a dashed line). The number of explants per condition is indicated on the bars. * = $p < 0.05$ compared to control IgG.

Scale bar: 200 μm.

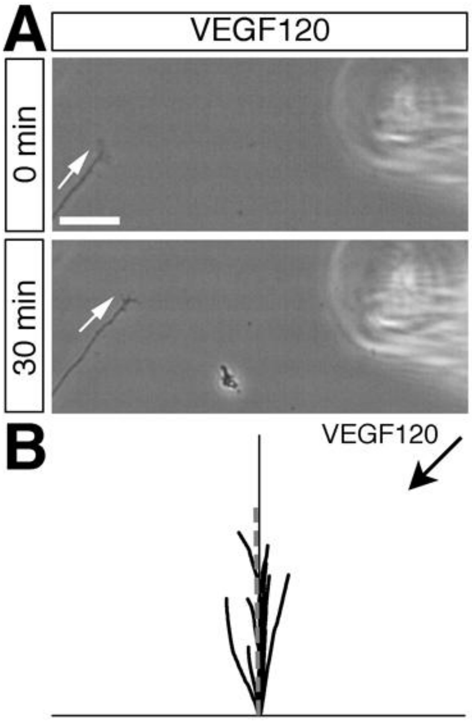

Supplemental Figure 5, related to Figure 7. VEGF120 does not evoke a guidance response in RGC growth cones.
(A) RGC growth cones at 0 min and after 30 min of exposure to a gradient of VEGF120; white arrows indicate the direction of growth cone extension.
(B) Superimposed RGC axon trajectories over the 30 min observation period; black arrows indicate the direction of the gradient.
Scale bar: 25 µm.

SUPPLEMENTAL EXPERIMENTAL PROCEDURES

Mouse strains

All animal procedures were performed in accordance with institutional and UK Home Office guidelines. Mice were mated in the evening, and the morning of vaginal plug formation was counted as embryonic (E) 0.5 days. To stage-match embryos, we compared facial and limb development. *Nrp1*-null mutants were obtained by breeding heterozygous mice on a JF1 genetic background to heterozygous mice on a CD1 background to extend the viability of *Nrp1*-null mutants beyond E12.5 (Kitsukawa et al., 1997; Schwarz et al., 2004). We also used mice carrying *Nrp2-null* or conditional *Nrp1* null alleles ($Nrp1^{fl/fl}$) and mice with mutations that disrupt semaphorin-signalling through NRP1 ($Nrp1^{Sema-/-}$)(Giger et al., 2000; Gu et al., 2003). To delete NRP1 in endothelial cells, we mated $Nrp1^{fl/fl}$ mutants to heterozygous *Nrp1*-null mice carrying a $Tie2^{Cre}$ transgene (Gu et al., 2003; Kisanuki et al., 2001). Mice lacking VEGF164 and VEGF188, but expressing VEGF120 ($Vegfa^{120/120}$; Carmeliet et al., 1999; Ruhrberg et al., 2002), carrying a *Sema3a* null allele (Taniguchi et al., 1997) and $Flt1^{LacZ}$ or $Flk1^{LacZ}$ reporters (Fong et al., 1995; Shalaby et al., 1995) have been described. Genotyping protocols can be supplied on request.

In situ hybridisation

In situ hybridisation was performed on 100 µm vibratome (Fig. 1-3, S2, S3) or 20 µm cryostat sections (Fig. 6) as described (Thompson et al., 2006a; Fig. 3C), using digoxigenin-labelled riboprobes for *Nrp1,* or *Nrp2* (Schwarz et al., 2008), *Sema3a-f* (gifts of Dr Marc Tessier-Lavigne, Genentech), *Vegf164* (Breier et al., 1992), *Ephb1 or ephrinb2* (Williams et al., 2003), *Zic2* (Hererra et al. 2003), *NrCAM* (Williams et al., 2006), *Flk1* (Millauer et al., 1993) or *Flt1* (Breier et al., 1995). Images were captured on a Nikon SMZ1500 or Zeiss Axiophot stereomicroscope with a Nikon DXM1200 digital camera and ACT-1 software.

Immunofluorescence

Mouse embryo heads were fixed in 4% formaldehyde in PBS. Fixed tissue was cryopreserved in 30% sucrose/PBS and embedded in OCT to cut 15 μm cryosections or in 3% agarose to cut 100 μm vibratome sections. Immunostaining was performed as described (Erskine et al., 2000; Thompson et al., 2006b) with the following primary antibodies: mouse monoclonal anti-SSEA-1 (MC-480; dilution 1:9), anti-radial glia (RC2; 1:9), anti-ISL1/2 (39.4D5; 1:50) or anti PAX6 (1:400), all from the Developmental Studies Hybridoma Bank; rabbit anti-phosphohistone-H^3 (1:100) and mouse anti-BRN3A (1:300) from Millipore. To double label for NRP1 and neurofilament, we used a rabbit anti-neurofilament antibody (1:250; AB1987 from Millipore) together with goat anti-rat NRP1 (1:100; AF566 from R&D systems), followed by Alexafluor488-conjugated goat anti-rabbit IgG (Invitrogen) and Cy3-conjugated donkey anti-goat Fab fragment (Jackson Immunoresearch). To double label for NRP1 and blood vessels, we used goat anti-rat NRP1 together with biotinylated isolectin B4 (IB4; Sigma), followed by Cy3-conjugated donkey anti-goat Fab fragment and Alexafluor488-conjugated streptavidin (Invitrogen). Slides were mounted in 90% glycerol/PBS or Vectashield (Vector Labs) and imaged using a Nikon SMZ1500 and DXM1200 camera or a Zeiss LSM510 confocal microscope.

Anterograde and retrograde DiI labelling

Anterograde DiI labelling was performed as described (Plump et al., 2002; Thompson et al., 2006a; Fig. S2A). NIH Image was used to measure the fluorescent intensity of the ipsilateral and contralateral optic tracts in non-saturated wholemount images (Fig. 2D). The ipsilateral index was calculated by dividing the fluorescent intensity in the ipsilateral optic tract by the sum of the fluorescent intensity in both tracts (adaptation of the method in Herrera et al., 2003). Wild type samples from different genetic backgrounds yielded similar

results, demonstrating reproducibility (compare Fig. 2D and 4B,D,F). Retrograde labelling of VEGF164 mutants yielded comparable results, further validating this approach (compare Fig. 4F and 5C). Retrograde DiI labelling was performed as described previously (Manuel et al., 2008). Briefly, DiI crystals were placed in a row over the dorsal thalamus on one side of the brain of formaldehyde-fixed embryos and incubated for 6-9 weeks at room temperature (Fig. 5A). In some experiments, retinas were flatmounted in Vectashield and photographed using a Nikon SMZ1500 microscope and DXM1200 camera. To quantify the relative size of the ipsilateral projection, we determined the number and distribution of labelled RGCs in all 200 µm horizontal sections through the entire contralateral and ipsilateral retina. Statistical comparisons were performed using the Mann Whitney U-Test.

RGC explant cultures

Peripheral retina from E14.5 C57 BL/6J was explanted into a 1:1 mixture of bovine dermis and rat tail collagen (BD Biosciences) or onto glass-bottomed dishes (MatTek Corporation) coated with poly-ornithine (Sigma-Aldrich) and 10 µg/ml laminin (Invitrogen), as described (Erskine et al., 2000; Williams et al., 2003). PBS, 10 or 50 ng/ml recombinant VEGF164 or VEGF120 in PBS was added to the culture medium comprised of DMEM:F12 (Invitrogen), 1% BSA and ITS supplement (Sigma-Aldrich). In some experiments, 0.5 µg/ml function-blocking goat anti-rat NRP1 (AF566 from R&D systems; neutralisation dose ND_{50} 0.3-1 µg/ml), 0.3 µg/ml function blocking goat anti-rat FLK1/VEGFR2 antibody (AF644 from R&D systems; ND_{50} 0.1-0.3 µg/ml) or 1 µg/ml control goat IgG (R&D systems) was included. After 24 h, the cultures were fixed and stained with mouse anti-β-tubulin antibody (1:500; Sigma-Aldrich) followed by Cy3-conjugated goat anti-mouse IgG (1:2000; Jackson ImmunoResearch) and photographed using a Nikon SMZ1500 microscope and DXM1200 camera. Image J (http://rsbweb.nih.gov/ij/) was used to quantify the area covered by the RGC axons as a measure of total axon outgrowth in a minimum of 3 independent

experiments. For this analysis, we deleted the explant core from the images, converted the remainder of the image containing the axons that had grown out of the explant into binary mode and quantified the number of black pixels. 1 or 2 explants per experiment lacked outgrowth completely and were excluded from the analysis. We also measured the area of each explant to ensure that differences in explant size did not affect our quantiation, but found no significant differences between control and VEGF-treated cultures. Statistical comparisons were made using ANOVA or the Mann Whitney U-test.

Growth cone turning assay

Growth cone turning assays were performed using an adaptation of the method of Murray and Shewan (2008). Ventrotemporal or dorsotemporal retinal explants were cultured on laminin as above. After 24 h, the dish was flooded with medium warmed to 37˚C and overlaid with a thin layer of vegetable oil. Individual growth cones were positioned on a heated microscope stage at a 45˚ angle and 100 µm distance from a micropipette containing PBS, VEGF164 (50 µg/ml) or VEGF120 (50 µg/ml). They were imaged at 10 min intervals over a 30 min period using a Nikon Diaphot inverted microscope connected to a PC running QWin version 2.1 software (Leica). Reagent gradients were generated by continuous injection from the pipette by an air pulse of 3 psi at 2 Hz and 10 ms duration, applied with a Picospritzer III (Intracel). This resulted in a VEGF concentration of ~50 ng/ml at the growth cone (Lohof et al., 1992). In some experiments, we added 0.5 µg/ml function-blocking goat anti-rat NRP1 or control goat IgG to the culture medium, as in the outgrowth assays. The angle turned by the growth cone and the extent of axon outgrowth was calculated using Image J. Only growth cones that advanced more than 10 µm during the 30 min observation period were included in the analysis. For each condition, we collected data from a minimum of 9 growth cones from at least 3 independent experiments. Statistical comparisons were made using a Mann-Whitney U Test.

SUPPLEMENTAL REFERENCES

Breier, G., Albrecht, U., Sterrer, S., and Risau, W., (1992). Expression of vascular endothelial growth factor during embryonic angiogenesis and endothelial cell differentiation. Development *114*, 521-532.

Breier, G., Clauss, M., and Risau, W., (1995). Coordinate expression of vascular endothelial growth factor receptor-1 (flt-1) and its ligand suggests a paracrine regulation of murine vascular development. Dev. Dyn. *204*, 228-239.

Carmeliet, P., Ng, Y.S., Nuyens, D., Theilmeier, G., Brusselmans, K., Cornelissen, I., Ehler, E., Kakkar, V.V., Stalmans, I., Mattot, V., Perriard, J.C., Dewerchin, M., Flameng, W., Nagy, A., Lupu, F., Moons, L., Collen, D., D'Amore, P.A. and Shima, D.T., (1999). Impaired myocardial angiogenesis and ischemic cardiomyopathy in mice lacking vascular endothelial growth factor isoforms VEGF164 and VEGF188. Nat. Med. *5*, 495-502.

Fong, G.H., Rossant, J., Gertsenstein, M., and Breitman, M.L. (1995). Role of the Flt-1 receptor tyrosine kinase in regulating the assembly of vascular endothelium. Nature *376*, 66-70.

Giger, R.J., Cloutier, J.F., Sahay, A., Prinjha, R.K., Levengood, D.V., Moore, S.E., Pickering, S., Simmons, D., Rastan, S., Walsh, F.S., Kolodkin, A.L., Ginty, D.D., and Geppert, M., (2000). Neuropilin-2 is required in vivo for selective axon guidance responses to secreted semaphorins. Neuron *25*, 29-41.

Kisanuki, Y. Y., Hammer, R.E., Miyazaki, J., Williams, S.C., Richardson, J.A. and Yanagisawa, M., (2001). Tie2-Cre transgenic mice: a new model for endothelial cell-lineage analysis in vivo. Dev. Biol. *230*, 230-242.

Kitsukawa, T., Shimizu, M., Sanbo, M., Hirata, T., Taniguchi, M., Bekku, Y., Yagi, T., and Fujisawa, H. (1997). Neuropilin-semaphorin III/D-mediated chemorepulsive signals play a crucial role in peripheral nerve projection in mice. Neuron *19*, 995-1005.

Millauer, B., Wizigmann-Voos, S., Schnurch, H., Martinez, R., Moller, N.P., Risau, W., and Ullrich, A., (1993). High affinity VEGF binding and developmental expression suggest Flk-1 as a major regulator of vasculogenesis and angiogenesis. Cell *72*, 835-846.

Shalaby, F., Rossant, J., Yamaguchi, T.P., Gertsenstein, M., Wu, X.F., Breitman, M.L., and Schuh, A.C. (1995). Failure of blood-island formation and vasculogenesis in Flk-1-deficient mice. Nature *376*, 62-66.
